# A Future Vision for *PLOS Computational Biology*

**DOI:** 10.1371/journal.pcbi.1002727

**Published:** 2012-10-04

**Authors:** Ruth Nussinov

**Affiliations:** 1 Basic Science Program, SAIC-Frederick, Inc., Center for Cancer Research Nanobiology Program, Frederick National Laboratory for Cancer Research, National Cancer Institute, Frederick, Maryland, United States of America; 2 Sackler Institute of Molecular Medicine, Department of Human Genetics and Molecular Medicine, Sackler School of Medicine, Tel Aviv University, Tel Aviv, Israel

With much trepidation I accepted the great honor and responsibility of becoming Editor-in-Chief of *PLOS Computational Biology*. I am fully aware of how hard it will be to step into the shoes of Philip Bourne, the Editor-in-Chief of the journal for the last seven years, since it was founded by him, Steven Brenner, and Michael Eisen. We are all deeply appreciative and thankful to Phil for his unique role; and we are grateful that he will be continuing his association with the journal in the future in the role of Founding Editor-in-Chief, aiding and inspiring us and the *PLOS Computational Biology* community. As a Deputy Editor-in-Chief, I became aware of the true family relationship in the broad PLOS organization and of the devoted and gifted editorial force so nicely fostered by Phil in *PLOS Computational Biology*. These played a crucial role in helping me decide to accept the invitation to become Editor-in-Chief.

I am deeply committed to the underlying principle of free public access to scientific information. In particular, what is truly unique and special about *PLOS Computational Biology* is that it fulfills this mission while maintaining the highest standards of scientific rigor, originality, and clear biological relevance. As Editor-in-Chief I will do my best to have *PLOS Computational Biology* continue these traditions.

Computational biology is often perceived as a single field; this however is not the case. Like experimental biology, computational biology is enormously broad; the only distinction from experimental biology is the means. This has disadvantages and advantages: the main disadvantage is that conclusions based on computations are often treated by biologists as less conclusive than those based on experiments; the main advantages are that computations allow researchers to analyze vast amounts of data and make testable predications, and they allow researchers to address problems that current experimental methods may not be able to treat. The high quality of papers published in *PLOS Computational Biology* indicates that the apparent disadvantage is not necessarily there. While they may still need further experimental and computational validation, conclusions based on rigorous computations applied to carefully assembled and curated data, which are backed up by available experimental results, can be as reliable, insightful, and biologically significant as those based on experiments.

*PLOS Computational Biology* is broad; it addresses diverse biological problems. We look forward to further expanding its scope through the inclusion of outstanding methods and resource papers, opening up significant new research directions while retaining and enhancing the strong scientific merit of *PLOS Computational Biology* publications. We further plan on improving the pace of submissions processing. *PLOS Computational Biology* is recognized by the community as the premier journal in computational biology. We will strive to continue in this tradition.

*PLOS Computational Biology* is tightly associated with the International Society of Computational Biology (ISCB). We cherish and will continue fostering this association. The Society, its meetings, and the journal all have a common goal: enhancing and promoting excellence in computational biology. Outstanding research with clear biological relevance, which leads to fundamental new insights into important biological problems, is the hallmark of future contributions by our community to biology. As the Editor-in-Chief I shall do my utmost to achieve these goals.

